# On the Performance Evaluation of a MIMO–WCDMA Transmission Architecture for Building Management Systems

**DOI:** 10.3390/s18010155

**Published:** 2018-01-08

**Authors:** Eleftherios Tsampasis, Panagiotis K. Gkonis, Panagiotis Trakadas, Theodοre Zahariadis

**Affiliations:** Department of Electrical Engineering, School of Technological Applications, Technological Educational Institute of Sterea Ellada, 344 00 Dirfies Messapies, Greece; pgonis@teiste.gr (P.K.G.); ptrakadas@teiste.gr (P.T.); zahariad@teiste.gr (T.Z.)

**Keywords:** building management systems, wideband code division multiple access (WCDMA), multiple input–multiple output (MIMO), Monte Carlo simulations, performance evaluation

## Abstract

The goal of this study was to investigate the performance of a realistic wireless sensor nodes deployment in order to support modern building management systems (BMSs). A three-floor building orientation is taken into account, where each node is equipped with a multi-antenna system while a central base station (BS) collects and processes all received information. The BS is also equipped with multiple antennas; hence, a multiple input–multiple output (MIMO) system is formulated. Due to the multiple reflections during transmission in the inner of the building, a wideband code division multiple access (WCDMA) physical layer protocol has been considered, which has already been adopted for third-generation (3G) mobile networks. Results are presented for various MIMO orientations, where the mean transmission power per node is considered as an output metric for a specific signal-to-noise ratio (SNR) requirement and number of resolvable multipath components. In the first set of presented results, the effects of multiple access interference on overall transmission power are highlighted. As the number of mobile nodes per floor or the requested transmission rate increases, MIMO systems of a higher order should be deployed in order to maintain transmission power at adequate levels. In the second set of results, a comparison is performed among transmission in diversity combining and spatial multiplexing mode, which clearly indicate that the first case is the most appropriate solution for indoor communications.

## 1. Introduction

Modern trends in the design of building facilities include minimum energy usage combined with a holistic monitoring of building infrastructures in terms of energy consumption. In this context, not only can consumed energy be reduced but also an environmental framework for energy management can be established in the long term [1]. This concept is also referred to as building management system (BMS), which includes a variety of diverse operations, from sensing and wireless transmission to decision making procedures and building automations. Typical responsibilities of a BMS include, but are not limited to, video surveillance systems and lighting, monitoring air quality (temperature, humidity, carbon monoxide, carbon dioxide, hydrogen sulfide, ozone, and particles), indicating when the air quality is not proper for living (ventilation), monitoring energy quality and quantity (e.g., current, voltage, active energy, active power, water, and gas), measurements of inside and outside temperature, and humidity measurements [2]. 

In the near future, it is expected that BMS protocols and operations will be incorporated in fifth-generation (5G) mobile communications, thus enhancing user experience in smart building management [3]. In addition, BMSs should also allow bidirectional communication with smart grid (SG) infrastructure [4,5] in order to accurately predict energy consumption and demand. Therefore, appropriate BMS deployment requires a reliable network infrastructure able to gather and process information under various conditions. In this context, a well-known and established practice is the deployment of wireless sensor networks (WSNs) on the premises of the building, properly installed in order to measure a variety of values and report back to the central server. Proper installation should take into account minimization of overall transmission power of wireless nodes as well as indoor propagation that can significantly deteriorate signal quality.

Several approaches in recent literature have attempted to provide an insight into modern BMS trends. In [6], load control and load balancing techniques are investigated, in the concept of artificial intelligence in BMS orientations. In [7], an initial attempt to create a secure data-centric BMS architecture is presented. To this end, a hierarchical namespace across application data and user identities to simplify user authentication and data access control are presented. In [8], various protocols are analyzed for BMS applications. The authors conclude that KNX, LonWork, and BACnet protocols [9] are appropriate for building automation because of their high safety, adaptability with other products, reasonable prices, and simple installation. In the same context, and in order to overcome the heterogeneity of various building protocols, in [10], the authors propose the Web-of-Things (WoT) framework, using well-known standard technologies of the Web such as HTTP and RESTful APIs, for standardizing the access to devices seen from an application point of view. In [11], a flexible WoT hierarchical architecture model is presented for the concept of smart cities and aims to interconnect various diverse technologies. In [12], the BMS concept for buildings with a large-scale automation system is presented. The basic idea was to build a control system database as an extension of the Building Information Modeling (BIM) database, which contains the definition of an automation system and a technological infrastructure controlled by that automation.

Other works have focused on the performance evaluation of communication protocols for wireless sensor networks. In [13], various medium access control (MAC) protocols for synchronous/asynchronous and single/multi-channel WSNs are investigated. Single-channel MAC protocols are categorized into synchronous and asynchronous approaches, and the advantages and disadvantages of each protocol are presented. In addition, the different features required in multi-channel WSNs compared to single-channel WSNs are investigated as well. In [14], specific output metrics in WSNs such as optimum distance among nodes as well as transmission power are extracted for the case of non-fading channels, considering specific modulation schemes.

**Figure 1:**
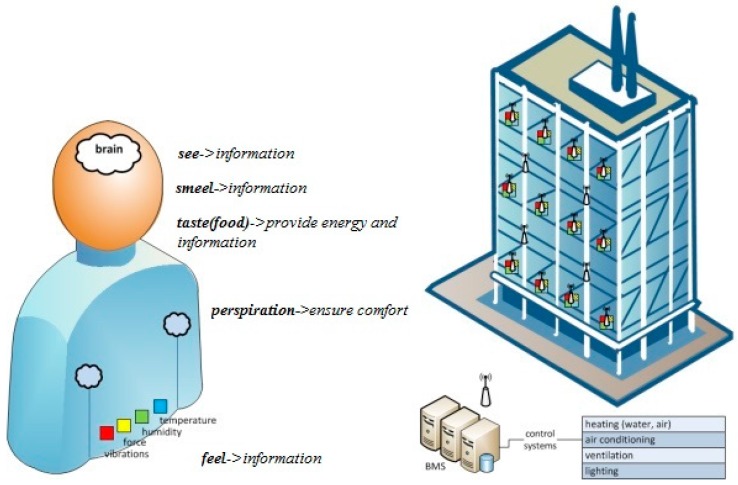
Typical building management system (BMS) infrastructure.

The goal of the work presented in this paper is to investigate the performance of a BMS orientation, where multiple input–multiple output (MIMO)—wideband code division multiple access (WCDMA) transmission and reception is adopted for the communication with network nodes, due to its inherent capability to take advantage of multipath propagation and minimize overall transceiver complexity [15]. With respect to Figure 1, each node is equipped with a multi-antenna system and a 2-D space–time rake receiver that estimates and coherently combines multipath replicas of the transmitted signal. Unlike other works with main emphasis in networking protocols and performance analysis considering a fixed node topology, our work not only covers ad-hoc cases but also focuses on the deployment of MIMO architecture in wireless nodes in order to optimize transmission power and improve network performance without additional spectrum requirements. In this context, it is assumed that node locations may vary according to traffic conditions in the building or other external factors; therefore, Monte Carlo (MC) simulations are performed where at each iteration a different network topology is considered. As integration with 5G standardization and SG infrastructure progresses [16], a physical layer protocol analysis is essential as well, since network nodes will play a dual role: measuring critical parameters and informing the BMS of appropriate actions as previously mentioned, as well as interacting with mobile users in order to provide information on demand.

The rest of this manuscript is organized as follows: In Section 2, the mathematical framework for wireless transmission in building environments is provided, with emphasis on multipath exploitation, considering a MIMO–3G infrastructure. Indoor propagation is considered, while performance metrics, such as per frame signal-to-interference-plus-noise ratio (SINR) and transmission power, are derived as well. In Section 3, the simulation framework is provided, while in Section 4 results are presented for various MIMO orientations (i.e., 2 × 2, 3 × 3, and 4 × 4) and six resolvable multipath components in terms of mean transmission power per mobile node. Finally, concluding remarks and proposals for future work are given in Section 5. 

## 2. MIMO–WCDMA Orientation 

A building orientation with *M* floors is considered, with *M_i_* sensor nodes per floor (1 ≤ *i* ≤ *M*). It is assumed that all nodes can communicate wirelessly with the central BMS. This is a rather realistic assumption, since we consider a dynamic environment where all nodes can be placed ad hoc. In order to reduce overall transmission power, each node is equipped with *M_t_* antennas, the central BS with *M_r_* antennas, while the WCDMA physical layer protocol has been adopted for internode communications [17]. In WCDMA, which is already used by mobile terminals in 3G communications, all nodes can transmit simultaneously occupying the whole transmission bandwidth. This is achieved with the use of orthogonal spreading sequences, which multiply the information signal. These sequences have low cross correlation values, thus minimizing unwanted effects from multipath propagation. The transmitted WCDMA signal for diversity combining transmission mode (i.e., the same signal is send and received from all antennas) is given by [17]

(1) $$x_{n}\left(t\right)=\sqrt{p_{n}}b_{n}\left(t\right)c_{n}\left(t\right)w_{n}$$

where *p_n_* is the power of the *n*th node (1 ≤ *n* ≤ *N*), *c_n_*(*t*) the coding sequence, and *b_n_*(*t*) the information signal. 

For *M*_t_ antennas at the transmitter, **w***_n_* represents the *M_t_* × 1 transmission vector that corresponds to the complex power distribution per antenna (i.e., $\|w_{n}\|{}_{F}^{2}$ = 1, where ${\left\|x\right\|}_{F}$ is the Frobenius norm of vector matrix **x**). It is assumed that *b_n_*(*t*) has a symbol duration equal to *T*, while the corresponding duration for the coding sequence is *T_c_*. The ratio *T*/*T_c_* is also called a spreading factor (*SF*). In a multipath environment, the received signal from the *M_r_* antennas of the central BS (considering uplink transmission) will be given by

(2) $$y_{n}\left(t\right)=\sqrt{\frac{p_{n}}{TL_{n}}}\sum_{l=1}^{L}H_{n,l}x_{n}\left(t-\tau_{n,l}\right)+\sqrt{\frac{p_{n\prime }}{TL_{n\prime }}}\sum_{\begin{array}{l} n\prime =1\\ n\prime \neq n \end{array}}^{N}\sum_{l=1}^{L}H_{n\prime ,l}x_{n\prime }\left(t-\tau_{n\prime ,l}\right)+n_{n}$$

where **y***_n_*(*t*) is the *M_r_* × 1 received signal vector matrix, *L* is the number of multipath components and *τ_l_* the corresponding delay of the *l*th multipath (1 ≤ *l* ≤ *L*). Moreover, the term *TL* denotes the total losses (i.e., due to pathloss, shadowing, antenna radiation patterns, etc.) from the transmitter to the receiver. Finally, **H***_n,l_* is the *M_r_* × *M_t_* channel matrix, and **n***_n_* the *M_r_* × 1 additive white Gaussian noise. In a rich scattering environment such as the inner of a building, each element of **H***_n,l_* is assumed to be a zero mean complex Gaussian random variable with standard deviation equal to one [18].

In order to exploit multipath propagation, it is assumed that each node is equipped with *L* rake receivers, also called fingers, which estimate the corresponding delay and coherently combine the individual components. The output from each finger will be given by [18]

(3) $$z_{n,l}=r_{n,l}\left(\begin{array}{l} \sqrt{\frac{p_{n}}{TL_{n}}}H_{n,l}w_{n}b_{n,0}+\\ \sqrt{\frac{p_{n}}{TL_{n}}}\sum_{\begin{array}{l} l\prime =1\\ l\prime \neq l \end{array}}^{L}H_{n,l\prime }w_{n}\left(\rho_{n,n,\left|l-l\prime \right|}b_{n,-1}+{\bar{\rho }}_{n,n,\left|l-l\prime \right|}b_{n,0}\right)+\\ \sqrt{\frac{p_{n\prime }}{TL_{n\prime }}}\sum_{\begin{array}{l} n\prime =1\\ n\prime \neq n \end{array}}^{N}\sum_{l\prime =1}^{L}H_{n\prime ,l\prime }w_{n\prime }\left(\rho_{n,n\prime ,\left|l-l\prime \right|}b_{n\prime ,-1}+{\bar{\rho }}_{n,n\prime ,\left|l-l\prime \right|}b_{n\prime ,0}\right)+n_{n} \end{array}\right)$$

In Equation (3), b*_n_*_,0_ is the symbol at the current time offset, while *b_n_*_,−1_ the corresponding symbol at the previous time offset. Moreover, **r***_n,l_* is the multiplying maximal ratio combining (MRC) vector matrix, given by [18,19]

(4) $$r_{n,l}={\left(H_{n,l}w_{n}\right)}^{H}$$

While *ρ* and $\bar{\rho }$ are the partial cross-correlations of the spreading sequences:

(5) $$\rho_{n,n\prime ,l}=\int_{0}^{lT_{c}}\bar{c_{n}(t-lT_{c})}c_{n\prime }(t)dt$$

(6) $${\bar{\rho }}_{n,n\prime ,l}=\int_{lT_{c}}^{Τ}\bar{c_{n}(t-lT_{c})}c_{n\prime }(t)dt$$

where $\bar{x}$ is the conjugate of x. The desired signal power will be given by

(7) $$P_{s,n}=\frac{p_{n}}{TL_{n}}w_{n}^{H}\left({\left(\sum_{l=1}^{L}r_{n,l}H_{n,l}\right)}^{H}\left(\sum_{l=1}^{L}r_{n,l}H_{n,l}\right)\right)w_{n}$$

while multiple access interference (MAI) from the *n*’th node (*n*’ ≠ *n*), intersymbol interference (ISI), and noise power will be given by Equations (8)–(10), respectively:

(8) $$\begin{array}{c} P_{ISI,n}={\left|\sum_{l=1}^{L}\sum_{\begin{array}{c} l\prime =1\\ l\prime \neq l \end{array}}^{L}\left(r_{n,l}H_{n,l\prime }w_{n}\right)\sqrt{\frac{p_{n}}{TL_{n}}}\left(\rho_{n,n,|l-l\prime |}+{\bar{\rho }}_{n,n,|l-l\prime |}\right)\right|}^{2}+\\ \sum_{l=1}^{L}{\left(r_{n,l}H_{n,l}w_{n}\sqrt{\frac{p_{n}}{TL_{n}}}\right)}^{H}\sum_{l=1}^{L}\sum_{\begin{array}{l} l\prime =1\\ l\prime \neq l \end{array}}^{L}\left(r_{n,l}H_{n,l\prime }w_{n}\sqrt{\frac{p_{n}}{TL_{n}}}\right)\left(\rho_{n,n,|l-l\prime |}+{\bar{\rho }}_{n,n,|l-l\prime |}\right)+\\ \sum_{l=1}^{L}\left(r_{n,l}H_{n,l}w_{n}\sqrt{\frac{p_{n}}{TL_{n}}}\right){\left(\sum_{l=1}^{L}\sum_{\begin{array}{l} l\prime =1\\ l\prime \neq l \end{array}}^{L}\left(r_{n,l}H_{n,l\prime }w_{n}\sqrt{\frac{p_{n}}{TL_{n}}}\right)\left(\rho_{n,n,|l-l\prime |}+{\bar{\rho }}_{n,n,|l-l\prime |}\right)\right)}^{H} \end{array}$$

(9) $$P_{MAI,n\prime }=\left({\left|\sum_{l=1}^{L}\sum_{l\prime =1}^{L}\left(r_{n,l}H_{n,l\prime }w_{n}\right)\left(\rho_{n,n\prime ,|l-l\prime |}+{\bar{\rho }}_{n,n\prime ,|l-l\prime |}\right)\right|}^{2}\right)\frac{p_{n\prime }}{TL_{n\prime }}$$

(10) $$P_{noise,n}=N_{o}\sum_{l=1}^{L}{\left\|r_{n,l}\right\|}_{F}^{2}$$

where *N_o_* is the thermal noise level. Therefore, overall signal-to-interference-plus-noise ratio (SINR) for the *n*th node is formulated as follows:

(11) $$SINR_{n}=\frac{P_{s,n}}{P_{ISI,n}+\sum_{n\prime =1,n\prime \neq n}^{N}P_{MAI,n\prime }+P_{noise,n}}$$

Depending on channel conditions and network load, capacity is upper limited either by the soft or hard mechanism. Soft capacity is defined as the number of nodes for which the SINR criterion (i.e., SINR > *SINR*th, where *SINR*th is a given threshold for a specific type of service) is satisfied without power outage. On the other hand, hard capacity equals to the available number of spreading sequences in a given service area.

Pathloss propagation among different floors is modeled according to ITU-R specifications, where *v* is the distance power loss coefficient, *f* is the frequency in MHz, *d* is the separation distance in meters between the central BS and a mobile node, *L_f_*(*n*) is the floor penetration loss factor in dB, and *n* is the number of floors between central BS and mobile node. Considering propagation at 2 GHz, for the considered office environment, *v* = 3 and *L_f_*(dB) = 15 + 4 (*n* – 1). Hence, net pathlosses and shadowing effect are calculated as follows [20]:

(12) $$L_{\mathrm{total}}=20log_{10}\left(f\right)+vlog_{10}\left(d\right)+L_{f}\left(n\right)\;TL=L_{\mathrm{total}}+X_{\sigma }$$

where *X*_σ_ is a log-normally distributed random variable corresponding to shadowing effects. 

In a realistic mobile environment, uplink or downlink transmission power per node is updated periodically using SINR measurements. In this work, however, as we are mainly interested in capacity limitations stemming from the deployment of MIMO architecture, transmission powers are calculated after linear matrix inversion on the matrices that are derived from the deployment of the equivalent SINR criterion on all nodes. Note that Equation (8) can be alternately written by

(13) PISI,n=pnTLn{|∑l=1L∑l′=1l′≠lL(rn,lHn,l′wn)(ρn,n,|l−l′|+ρ‾n,n,|l−l′|)|2+2Re{∑l=1L(rn,lHn,lwn)H∑l=1L∑l′=1l′≠lL(rn,lHn,l′wn)(ρn,n,|l−l′|+ρ‾n,n,|l−l′|)}}

Hence, a linear form of equations can be derived for the *N* × 1 power vector matrix:

(14) Ap=B

In Equation (14), Matrices **A** (*n* × *n*) and **B** (*n* × 1) are given by

(15) A(n,n′)=−SINRthTLn′(|∑l=1L∑l′=1L(rn,lHn,l′wn)(ρn,n′,|l−l′|+ρ‾n,n′,|l−l′|)|2),n≠n′A(n,n)=1TLnwnH((∑l=1Lrn,lHn,l)H(∑l=1Lrn,lHn,l))wn−1TLn{|∑l=1L∑l′=1l′≠lL(rn,lHn,l′wn)(ρn,n,|l−l′|+ρ‾n,n,|l−l′|)|2+2Re{∑l=1L(rn,lHn,lwn)H∑l=1L∑l′=1l′≠lL(rn,lHn,l′wn)(ρn,n,|l−l′|+ρ‾n,n,|l−l′|)}}B=−SINRth1(N)No

where **1**(*N*) is an *N* × 1 matrix of ones.

## 3. Simulation Framework

**Figure 2:**
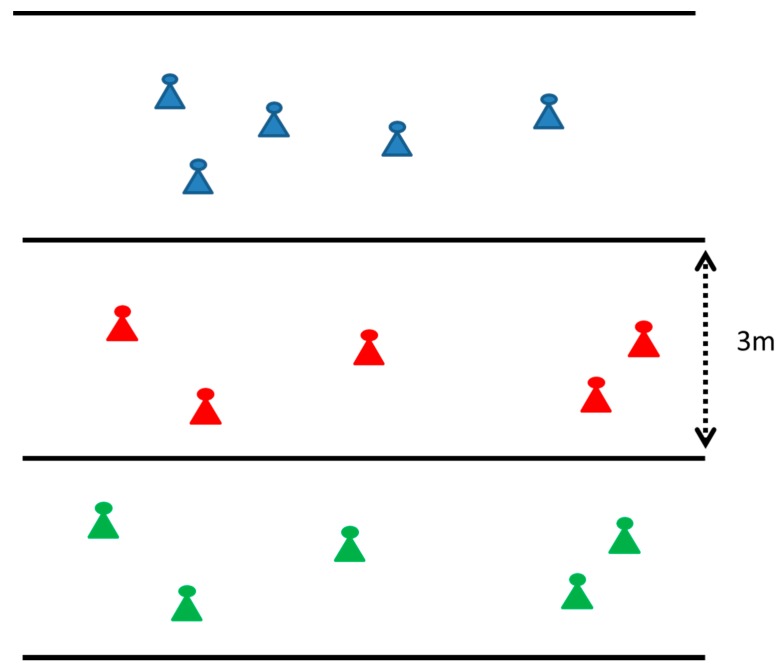
Wireless nodes in a three-floor building.

In this section, the simulation framework is provided considering a three-floor building with various sensor nodes per floor (Figure 2). Evaluation is performed with the help of MC simulations, where at each snapshot the nodes per floor can have a different orientation following a uniform distribution. At each snapshot, for every node in the network, the channel coefficient from the central BS is generated for all multipath components, according to the Gaussian distribution as described in Section 2. As explained, this is a rather realistic assumption, due to the number of reflections that take place inside a building. Once these coefficients are generated, corresponding parameters such as total losses are calculated per node. Afterwards, the matrix formulation according to Equations (14) and (15) takes place and overall transmission powers are calculated. 

As it will be explained in the following section, two transmission modes have been considered: diversity combining mode, where, as previously explained, the same information signal is sent and received from all transceiver antennas, and the spatial multiplexing transmission mode [21]. In the latter case, different information signals are sent from different transmit antennas. In this case, **w***_n_* is an *M_t_* × *M_t_* matrix. In both transmission cases, **w***_n_* matrices are calculated according to overall signal strength maximization. This iterative procedure is presented in Table 1 and Table 2, where tr(**X**) is the trace of Matrix **X**. In Table 1, **X**(*λ_m_*(**A**)) is the eigenvector corresponding to the maximum eigenvalue of Matrix **A**. In Table 2, **U** and **V** are the left and right singular matrices after singular value decomposition (SVD) of Matrix **A**, while **Σ** is a diagonal matrix containing the eigenvalues of **A** in descending order. Moreover, **I**_K_ is a K × K identity matrix, and diagonal matrix **D** indicates power distribution per transmission mode (tr(**D**) = 1). Finally, *dim* = min(*M_t_*,*M_r_*) indicates the dimension of the MIMO orientation. Both algorithms initially assume a uniform power distribution in all antennas, and at each step the desired signal strength is calculated. This procedure comes to an end once the convergence of both algorithms (defined by parameter *ε*) takes place. Finally, all simulation parameters are summarized in Table 3.

## 4. Results and Discussion

**Figure 3:**
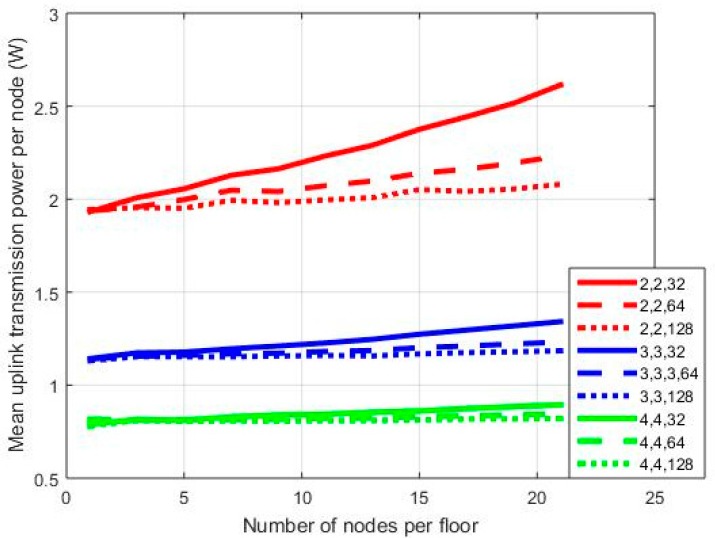
Mean power per node for SNR = 3 dB.

**Figure 4:**
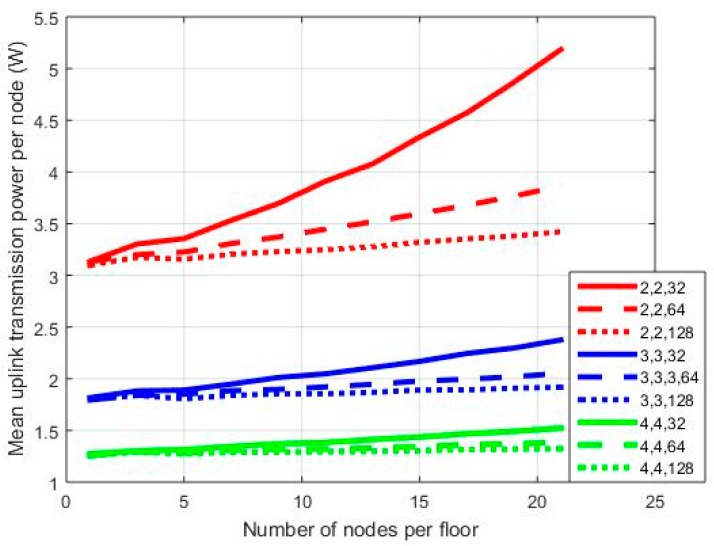
Mean power per node for SNR = 5 dB.

**Figure 5:**
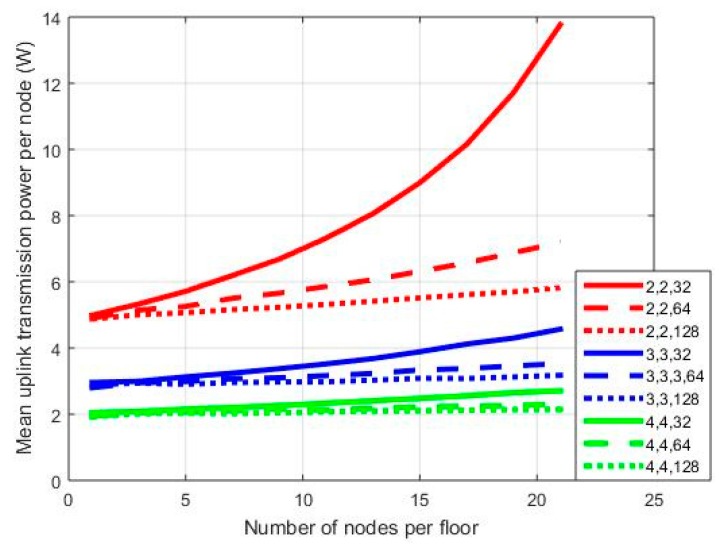
Mean power per node for SNR = 7 dB.

In Figure 3, Figure 4 and Figure 5, results are provided regarding the mean uplink transmission power of the network nodes to the BS for three MIMO orientations: 2 × 2, 3 × 3, and 4 × 4. Processing gain (*PG*) is equal to 128/64/32, while SNR may vary from 3 to 7 dB. In the first set of simulations, the diversity combining transmission mode has been assumed. Hence, for a WCDMA bandwidth equal to 3.84 MHz [1], the three *PG* values correspond to an equivalent transmission rate of 30/60/120 Kbps, respectively. 

In each graph, where mean transmission power per node is provided on a vertical axis, notation x,y,z stands for the number of transmit antennas/the number of receive antennas/*PG*. As can be observed in Figure 3, the mean transmission power is higher for the 2 × 2 orientation compared to the other two cases. With 21 nodes per floor and a *PG* equal to 128 in the 2 × 2 case, the mean transmission power is 2.08 W. The corresponding values for the 3 × 3 and 4 × 4 cases are 1.185 W and 0.8205 W, respectively. Hence, as the order of the MIMO orientation increases (i.e., the number of transmit and receive antennas), the mean transmission power decreases as expected, while this is also the case for the power reduction rate. Moreover, as can be observed from Figure 3, the 2 × 2 case is more vulnerable to *PG* variations. For *PG* = 64, the mean transmission power is 2.23 W, while the corresponding value for *PG* = 32 is 2.614 W. Hence, in the first case, we have an increment of 7% compared with the transmission power for *PG* = 128, and this increment further increases to almost 26% in the second case (i.e., *PG* = 32). When more antennas are added at both ends of the MIMO link, this increment is significantly lower, especially in the 4 × 4 orientation. In this case, for *PG* = 32 and one node per floor mean transmission power is 0.8159 W. For 21 nodes per floor, then this value increases to 0.8944 W. The corresponding values for the 2 × 2 case are 1.944 W/2.614 W, respectively. Hence, as the number of nodes increases, the MIMO order should be increased as well in order to calibrate transmission power increment.

In Figure 4 and Figure 5, the results corresponding to Figure 3 are presented for SNR = 5 dB and SNR = 7 dB, respectively. As can be observed in Figure 4, in the 2 × 2 case, the power gap among transmissions with *PG* = 128 and *PG* = 32 increased. For 21 nodes in the network, in the first case, the mean transmission power is 2.614 W, while in the latter case, it is 5.184 W; hence, there is an increment of almost 100%. In the 4 × 4 case, the corresponding transmission power is 0.8944 W/1.522 W for *PG* = 128/32, respectively. Hence, power increment is now 70%. Similar conclusions can be drawn from Figure 5 as well. Note, however, that in the 2 × 2 case there is a severe power outage for 21 nodes in the network. In all sets of simulations, no power thresholds were set, as the main goal was the performance evaluation of all MIMO orientations in extreme scenarios.

**Figure 6:**
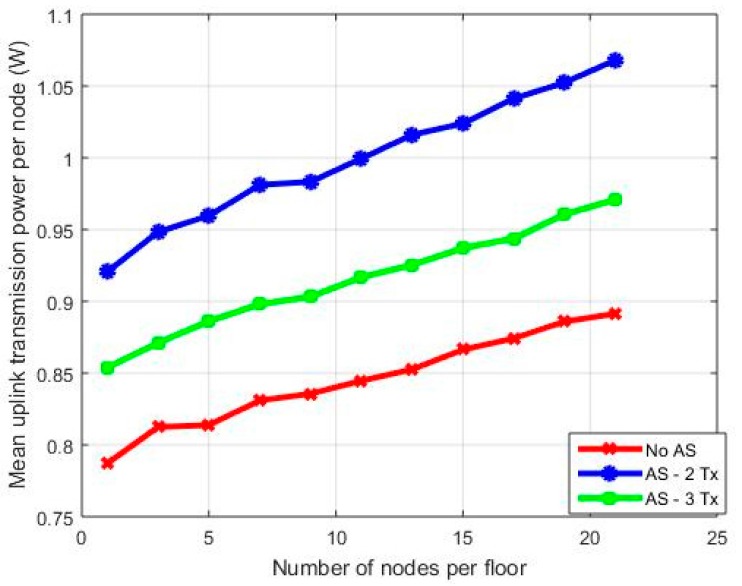
Mean power of nodes for various MIMO orientations with antenna selection—diversity combining transmission mode.

In Figure 6, results are provided for the case of antenna selection (AS) at mobile nodes. In this case, it is desirable to select a subset of transmit antennas in order to reduce RF complexity, at the cost, however, of increased transmission power. In the considered simulation scenario, a 4 × 4 MIMO transmission scenario has been considered with three cases: No AS, two selected antennas, and three selected antennas. Note that in the latter two cases, a subset of antennas is selected that maximizes the received SNR over all multipath components; hence,

(16) $$S_{n}\leftarrow \underset{j\in TR}{\arg\max}\|\overset{L}{\underset{l=1}{\prod }}H_{n,j,l}\|$$

where *S_n_* is the set of transmit antennas for the *n*th node, **H***_n_*_,*j*,*l*_ is the equivalent channel matrix considering transmission from the antennas in the subset *j*, and the set *TR* includes all possible combinations of transmit antennas. For example, considering a use case scenario with two selected transmit antennas, *TR* = {(1,2),(1,3),(1,4),(2,3),(2,4),(3,4)}.

As can be observed from Figure 6, as expected, transmission from all antennas is the best solution in terms of radiated transmitted power. For 21 nodes, when selecting three transmit antennas, the mean transmission power is almost 0.97 W, while the corresponding value for two transmit antennas is 0.89 W. Hence, compared to the case where no AS takes place, in the case of three transmit antennas, we have a power increment of almost 9%, which increases to 19% for the case of two transmit antennas. Note that AS burden is transferred to the BMS central BS, which gathers and processes all channel coefficients and then informs via feedback signals all nodes for the appropriate selection of transmit antennas.

In the final set of simulations, the spatial multiplexing transmission mode was considered. For a fair comparison with the previous cases of diversity combining, it is assumed that the input powers of the spatial multiplexing simulator are the ones that have been calculated at the diversity combining transmission mode, properly weighted. In this context, in the first case, a 2 × 2 MIMO orientation has been considered, while *PG* equals 64 in the diversity combining case and 128 in spatial multiplexing transmission mode. Note that, in both cases, the equivalent transmission rate is 60 Kbps, as in spatial multiplexing two different input streams are sent from the transmit antennas. In the second case, simulations are repeated for a 4 × 4 MIMO orientation, where now *PG* equals 32 in the diversity combining case and 128 in spatial multiplexing transmission mode.

**Figure 7:**
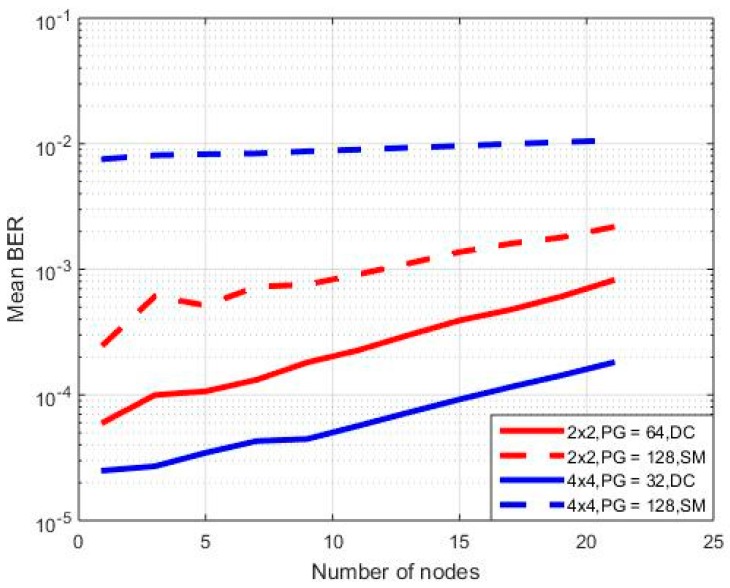
Mean bit error rate (BER) for various MIMO orientations—diversity combining and spatial multiplexing.

Results are presented in Figure 7, where mean bit error rate (BER) is plotted on a logarithmic scale for the aforementioned cases (DC stands for diversity combining and SM for spatial multiplexing. Details on theoretical BER calculations for spatial multiplexing transmission are provided in the Appendix). Note that, in this particular set of simulations, an SNR of 10 dB has been considered and a building orientation with one floor. Mobile nodes may vary from 1 to 21, as in the previous set of simulations, with a step of 2. Finally, the power per node in the spatial multiplexing cases (2 × 2 and 4 × 4) has been set to be double the value of the corresponding power in diversity combining mode for a 1 × 2/2 × 4 MIMO system, respectively, as now the diversity order of the system is reduced to half. As can be observed in Figure 7, in all cases of mobile nodes, spatial multiplexing transmission worsens the mean BER. This result is rather expected, as spatial multiplexing can be severely affected both by MAI and multiple reflections in indoor environments [22]. Note that BER in SM is affected to a greater extent in the 4 × 4 MIMO case, as MAI is the main capacity limiting factor due to the multiple antennas. This particular simulation scenario with four different input streams corresponds to four independent diversity combining cases (i.e., 1 × 4). Therefore, each receive antenna now undergoes a greater amount of interference, as it receives *M_t_* × *L* independent signals. Hence, diversity combining is the most appropriate solution for this type of communication, especially if we are interested in low-rate communications.

## 5. Conclusions

The performance of a MIMO–WCDMA architecture inside a building environment was analyzed. Two transmission modes (diversity combining and spatial multiplexing) were considered along with various amounts of nodes in a three-floor building. For each simulation scenario, a sufficient number of independent MC simulations were carried out for different MIMO orientations. According to the presented results, diversity transmission can improve the overall performance of the proposed approach, considering a multipath environment. In this context, high data rates along with an increased number of mobile nodes can be supported by increasing the order of the MIMO configuration. On the other hand, spatial multiplexing is not an efficient solution, as this type of transmission can be severely affected by multiple access interference. In contrast to wireless cellular networks, the number of nodes in indoor BMSs is known a priori. Therefore, for a specific uplink power threshold and transmission rate, an appropriate MIMO deployment can be selected, based on the extracted parametric curves (i.e., power versus nodes, SNR, number of transmit/receive antennas, antenna selection, transmission mode, etc.).

Ongoing work includes alternate transmission scenarios, such as the use of relay nodes and a cross-layer approach to interconnect the presented architecture with Internet of Things technology.

## Figures and Tables

**Table 1:** Maximization of the signal-to-noise ratio (SNR) in diversity combining mode.

**Step 1**: Set *i* ←1, $w_{n,i}\leftarrow \left(1/\sqrt{M_{t}}\right),P_{i}\leftarrow w_{n,i}^{H}w_{n,i}$ , *ε* = 10^−3^
**Step 2**: $r_{n,l,i}={\left(H_{n,l}w_{n,i}\right)}^{H},1\leq l\leq L\;\mathrm{and}\;A\leftarrow \sum_{l=1}^{L}\sum_{l\prime =1}^{L}{\left(r_{n,l,i}H_{n,l}\right)}^{H}r_{n,l\prime ,i}H_{n,l\prime }$
**Step 3**: $w_{n,i+1}\leftarrow X\left(\lambda_{m}\left(A\right)\right)P_{i+1}\leftarrow w_{n,i+1}^{H}Aw_{n,i+1}$ and $P_{i+1}\leftarrow w_{n,i+1}^{H}Aw_{n,i+1}$
**Step 4**: If $\frac{\left|\mathrm{tr}\left(P_{i+1}\right)-\mathrm{tr}\left(P_{i}\right)\right|}{\mathrm{tr}\left(P_{i}\right)}\geq \varepsilon$ go to Step 2

**Table 2:** Maximization of the SNR in spatial multiplexing transmission mode.

**Step 1**: Set *i* ←1, $w_{n,i}\leftarrow \frac{1}{\sqrt{M_{t}}}I_{dim}$, $P_{i}\leftarrow w_{n,i}^{H}w_{n,i}$, *ε* = 10^−3^
**Step 2**: $r_{n,l,i}={\left(H_{n,l}w_{n,i}\right)}^{H},1\leq l\leq L$ and $A\leftarrow \sum_{l=1}^{L}\sum_{l\prime =1}^{L}{\left(r_{n,l,i}H_{n,l}\right)}^{H}r_{n,l\prime ,i}H_{n,l\prime },A\leftarrow U\Sigma V^{H}$
**Step 3**: $w_{n,i+1}\leftarrow VD^{1/2}$ and $P_{i+1}\leftarrow w_{n,i+1}^{H}Aw_{n,i+1}$
**Step 4**: If $\frac{\left|\mathrm{tr}\left(P_{i+1}\right)-\mathrm{tr}\left(P_{i}\right)\right|}{\mathrm{tr}\left(P_{i}\right)}\geq \varepsilon$ go to Step 2

**Table 3:** Simulation parameters.

Parameter	Units	Value/Assumption
Frequency	MHz	2000
Total bandwidth	MHz	3.84
Number of floors		3
Standard deviation of shadow fading	dB	10
MC snapshots per simulation		10^6^
Multipath components (*L*)		6
Processing gain (*PG*)		32, 64, 128
Antenna radiation pattern per antenna		Omnidirectional
Number of antennas at the transmitter (*M_t_*)		2,3,4
Number of antennas at the receiver (*M_r_*)		2,3,4
Number of nodes per floor		1–21
*SINR_th_*	dB	3/5/7/10
Floor height (*F_h_*)	m	3
Transmission mode		Diversity combining/spatial multiplexing

**Figure A1:**
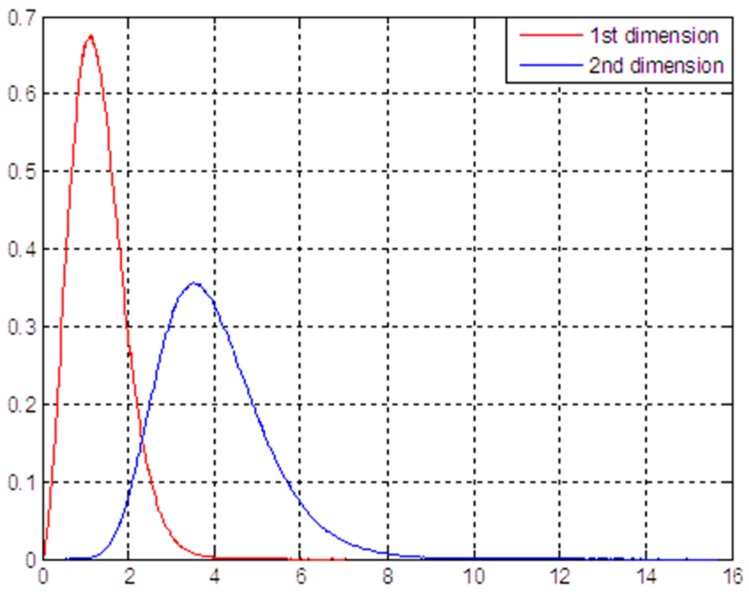
Gamma distributions of the curves in Equations (A8) and (A9).

**Figure A2:**
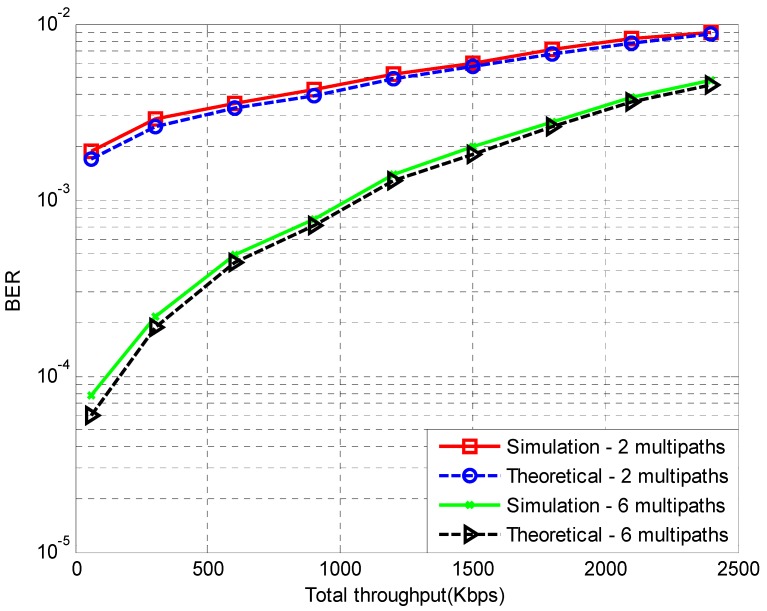
Simulation and theoretical BER for a 4 × 2 MIMO—WCDMA network.

**Figure A3:**
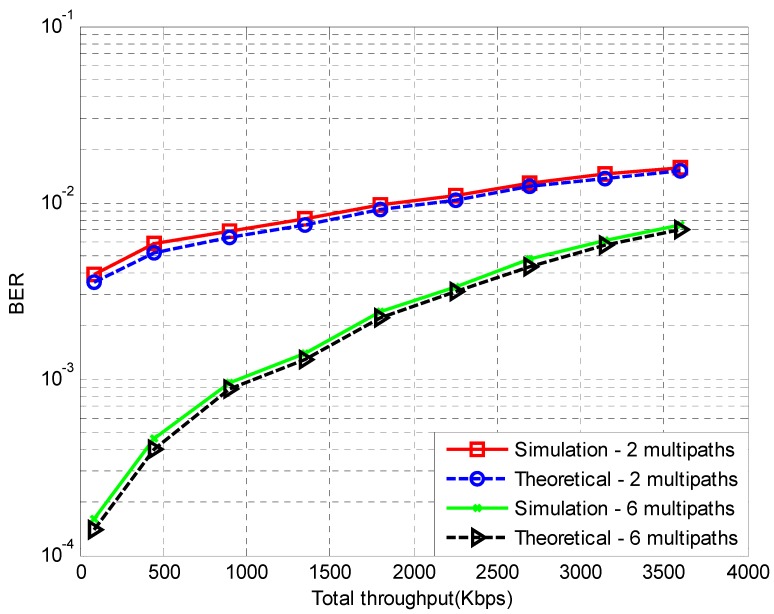
Simulation and theoretical BER for a 4 × 3 MIMO—WCDMA network.

**Figure A4:**
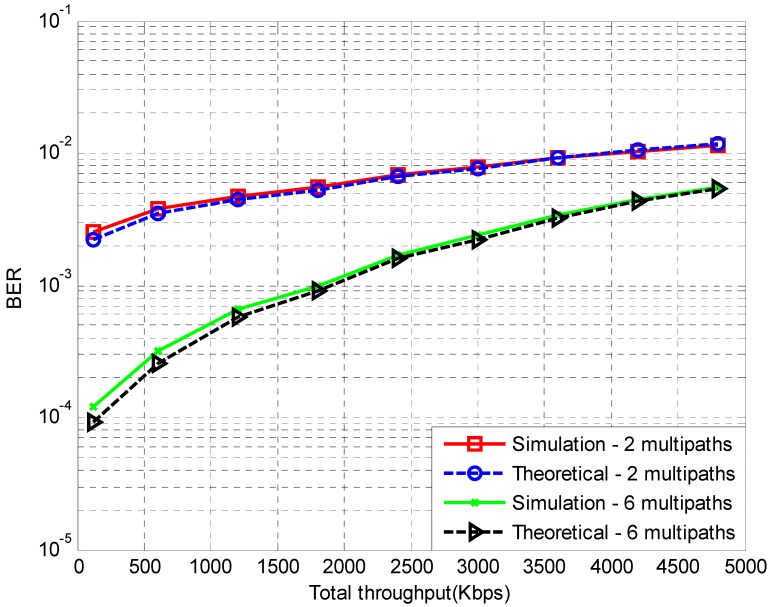
Simulation and theoretical BER for a 8 × 4 MIMO—WCDMA network.

## Appendix A

In the Gaussian approach for the theoretical calculation of the BER in the spatial multiplexing mode, the *n*th user is considered as the desired user, and MAI from the remaining *N*-1 users as well as ISI are expressed in terms of the desired user signal. With respect to Equation (9) [23],

(A1) $$E\left\{\overset{L}{\underset{l=1}{\sum }}\|r_{n,l}H_{n,l}w_{n\prime }\|{}_{F}^{2},d\right\}\to \overset{L}{\underset{l=1}{\sum }}\left\{\|r_{n,l}\|{}_{F}^{2},d\right\}p_{n^{\prime },d},\;1\leq d\leq dim\;$$

The term {**Α**,x} indicates the x^th^ line of Matrix **Α**. Moreover,

(A2) $$E\left\{\overset{L}{\underset{l=1}{\sum }}\|r_{n,l}H_{n,l}w_{n}\|{}_{F}^{2},d\right\}\to \overset{L}{\underset{l=1}{\sum }}{\left\{\|r_{n,l}\|{}_{F}^{2},d\right\}}^{2},1\leq d\leq dim$$

The SINR for the *n*th user and *d*th spatial multiplexing mode will be given by [23]

(A3) $$SINR_{n,d}\approx \frac{\sum_{l=1}^{L}\left\{\|r_{n,l}\|{}_{F}^{2},d\right\}p_{n,d}\;}{\left(N-1\right)\left(\frac{1}{6PG}\right)+\left(\frac{L-1}{L}\right)\left(\frac{1}{4PG}\right)+N_{0}},1\leq d\leq dim$$

In Equation (A3), *p_n,d_* is the transmission power per active user and mode, subject to

(A4) $$\overset{dim}{\underset{d=1}{\sum }}p_{n,d}=P_{n}\;$$

The desired user signal will be given by

(A5) $$\gamma_{n,d}=\overset{L}{\underset{l=1}{\sum }}\left\{\|r_{n,l}\|{}_{F}^{2},d\right\}p_{n,d}\;$$

For BPSK modulation, BER will be given by [18]

(A6) $$P_{e}=\frac{1}{dim}\overset{dim}{\underset{d=1}{\sum }}\int_{0}^{\infty }Q\left(\sqrt{SINR_{n,d}\left(\gamma_{n,d}\right)}\right)p_{\gamma_{n,d}}\left(\gamma_{n,d}\right)\;$$

where *P_e_* is the error probability, $p_{\gamma_{n,d}}\;\left(\gamma_{n,d}\right)$ is the probability density function (pdf) of the desired user signal of Equation (A5), and the Gaussian function is given by

(A7) $$Q\left(x\right)=\frac{1}{\sqrt{2\pi }}\int_{x}^{\infty }\exp\left(-\frac{u^{2}}{2}\right)du$$

In order to numerically evaluate the pdf of the desired signal per transmission mode, samples are taken from 10^7^ different channel realizations, where *γ_n_*_,*d*_ is calculated for each transmission mode. For a 4 × 2 MIMO network with six multipath components, the pdf functions of the two transmission modes follow the Gamma distribution [24]:

(A8) $$f_{1}\left(x\right)=x^{27}\frac{e^{-x/0.11}}{0.11^{28}\Gamma \left(28\right)}$$

(A9) $$f_{2}\left(x\right)=x^{18}\frac{e^{-x/0.09}}{0.09^{19}\Gamma \left(19\right)}$$

where Γ(x) = (x − 1)!, as shown in Figure A1. Simulation results are presented in Figure A2, Figure A3 and Figure A4. The mean BER is presented on a logarithmic scale versus throughput considering three different MIMO orientations: 4 × 2, 4 × 3, and 8 × 4, respectively, with two and six resolvable multipath components. An SNR equal to 10 dB is considered, as well as a uniform power delay profile regarding the individual powers of the multipath components. The number of active users may vary from 1 to 40 with a step of 5, so the total network throughput will vary from 30 × min(*M_t_*, *M_r_*) Kbps to 1200 × min(*M_t_*, *M_r_*) Kbps, i.e., from 60 to 2400 Kbps. Note that the basic transmission rate of 30 Kbps corresponds to a *PG* equal to 128. As can be observed in Figure A2, Figure A3 and Figure A4, the Gaussian approximation for the error probability and consequently the mean BER can be quite accurate for the performance evaluation of a MIMO–WCDMA system operating in spatial multiplexing mode. Moreover, the selection of all multipath components (i.e., 6) leads to an improved BER compared to the case of two multipath components. As expected, this gain reduces for high values of overall throughput, since for an increased number of active users, MAI can be significantly increased.
